# A case-control study of aggressiveness in adolescents with schizophrenia family history

**DOI:** 10.1192/j.eurpsy.2023.2268

**Published:** 2023-07-19

**Authors:** F. Ghrissi, F. Fekih-romdhane, M. Stambouli, B. Abassi, M. Cheour

**Affiliations:** psychiatry department E, Razi Hospital, Mannouba, Tunisia

## Abstract

**Introduction:**

Violence is a common behavioral and health concern among adolescents, aged 12 to 18 years old. In fact, aggressiveness may result in severe outcome in a critical age characterised by biological, psychological, and social changes. Schizophrenia is a severe and chronic condition, with elevated level of aggressiveness. Since unaffected biological relatives of schizophrenia patients share similar though less severe neurocognitive and behavioral abnormalities seen in their affected relatives, they are at increased risk of violence mainly during adolescence. However, studies including adolescents with schizophrenia first degree history are scarce.

**Objectives:**

The aim of this survey was to evaluate the aggressiveness among unaffected adolescents with fist degree family history of schizophrenia and in a control group of adolescents with no family psychiatric history.

**Methods:**

In this purpose wo conducted a case-control cross sectional study in Razi hospital during three months: from July to September 2022. Unaffected adolescents aged 12 to 18 whom first-degree relatives were diagnosed with schizophrenia according to DSM-5 criteria were included. Adolescents with psychiatric conditions or medical affections associated with psychiatric presentation were not included. Control group was selected amongst the population. Sociodemographic data were collected on a preestablished questionnaire and the following scales were used: The Life History of Aggression LHA, an 11 items self-reported tool, in the Arabic version, The Aggression Questionnaire AQ which is a 29 items self-reported scale in Arabic version. Written informed consent was obtained from the legal tutor of each adolescent.

**Results:**

Results of this survey are ongoing.

**Conclusions:**

Results of this survey are ongoing.

**Disclosure of Interest:**

None Declared

